# Neonatal admission and mortality in babies born in UK alongside midwifery units: a national population-based case-control study using the UK Midwifery Study System (UKMidSS)

**DOI:** 10.1136/archdischild-2020-319099

**Published:** 2020-10-30

**Authors:** Rachel Rowe, Aung Soe, Marian Knight, Jennifer J Kurinczuk, UK Midwifery Study System (UKMidSS), Mervi Jokinen

**Affiliations:** 1 National Perinatal Epidemiology Unit, Nuffield Department of Population Health, University of Oxford, Oxford, UK; 2 Oliver Fisher Neonatal Intensive Care Unit, Medway Maritime Hospital, Medway NHS Foundation Trust, Gillingham, Kent, UK

**Keywords:** epidemiology, neonatology

## Abstract

**Objectives:**

To determine the incidence of and risk factors for neonatal unit admission, intrapartum stillbirth or neonatal death without admission, and describe outcomes, in babies born in an alongside midwifery unit (AMU).

**Design:**

National population-based case-control study.

**Method:**

We used the UK Midwifery Study System to identify and collect data about 1041 women who gave birth in AMUs, March 2017 to February 2018, whose babies were admitted to a neonatal unit or died (cases) and 1984 controls from the same AMUs. We used multivariable logistic regression, generating adjusted OR (aOR) with 95% CIs, to investigate maternal and intrapartum factors associated with neonatal admission or mortality.

**Results:**

The incidence of neonatal admission or mortality following birth in an AMU was 1.2%, comprising neonatal admission (1.2%) and mortality (0.01%). White ‘other’ ethnicity (aOR=1.28; 95% CI=1.01 to 1.63); nulliparity (aOR=2.09; 95% CI=1.78 to 2.45); ≥2 previous pregnancies ≥24 weeks’ gestation (aOR=1.38; 95% CI=1.10 to 1.74); male sex (aOR=1.46; 95% CI=1.23 to 1.75); maternal pregnancy problem (aOR=1.40; 95% CI=1.03 to 1.90); prolonged (aOR=1.42; 95% CI=1.01 to 2.01) or unrecorded (aOR=1.38; 95% CI=1.05 to 1.81) second stage duration; opiate use (aOR=1.31; 95% CI=1.02 to 1.68); shoulder dystocia (aOR=5.06; 95% CI=3.00 to 8.52); birth weight <2500 g (aOR=4.12; 95% CI=1.97 to 8.60), 4000–4999 g (aOR=1.64; 95% CI=1.25 to 2.14) and ≥4500 g (aOR=2.10; 95% CI=1.17 to 3.76), were independently associated with neonatal admission or mortality. Among babies admitted (n=1038), 18% received intensive care. Nine babies died, six following neonatal admission. Sepsis (52%) and respiratory distress (42%) were the most common discharge diagnoses.

**Conclusions:**

The results of this study are in line with other evidence on risk factors for neonatal admission, and reassuring in terms of the quality and safety of care in AMUs.

## Abstract (precis)

Interesting national study of adverse events of babies born on UK midwifery units showing mortality and admission rate not raised (compared with obstetric units) and complications were uncommon.

**What is already known on this topic?**

- National guidance recommends that women at low risk of complications should have a choice of planned birth setting.
- For low-risk women planned midwifery unit birth is as safe for babies and associated with reduced intervention, compared with planned hospital obstetric unit birth.
- Neonatal admission following birth in a midwifery unit, where most women are low risk, not induced and give birth vaginally, is a potential ‘near-miss’ event.

**What this study adds?**

- Around 1% of babies born in the UK alongside midwifery units are admitted to neonatal care, are stillborn or die soon after birth without admission.
- Few babies had diagnoses or outcomes indicative of serious intrapartum-related adverse outcome.
- Risk factors for neonatal admission or mortality identified in our study are known risk factors for adverse neonatal outcome in term infants in other settings.

## Introduction

Most babies in high-income countries are born in hospital obstetric units (OU),^1 2^ but in the UK around 15% of births take place in midwifery-led settings.^3 4^ Around 80% of these are in alongside midwifery units (AMU), on the same site as an OU. For women at low risk of complications, planned birth in an AMU is associated with less intervention,^5^ including a 60% reduction in the likelihood of caesarean section, with no difference in neonatal outcomes, compared with planned birth in an OU.^6^ Most women planning birth in AMUs are at low risk, but around 4% have pre-existing risk factors^6 7^; many AMUs now explicitly admit women with selected risk factors.^8^ ^9^

National guidance recommends transfer to an OU when complications occur during labour in a midwifery-led setting.^10^ However, around 40% of adverse perinatal outcomes in births planned in midwifery-led settings occur in births in the planned setting, that is, when no transfer took place.^6^ Most babies admitted to neonatal care are born in OUs. Neonatal admission, or death without admission, following birth in an AMU, where most women are at low risk of complications, labour spontaneously and give birth vaginally, is a potential indicator of a ‘near-miss’ or adverse outcome event where different management might have made a difference to outcome. Factors associated with term admission and reasons for admission are well-documented,^11–15^ but there is no evidence about admission following birth in a midwifery-led setting. This study aimed to: (i) determine the incidence of and risk factors for admission to neonatal care, intrapartum stillbirth or neonatal death without admission, in babies born in an AMU and (ii) describe reasons for neonatal admission.

## Methods

### Study design

We carried out a national, population-based, case-control study.

### Cases and controls

We identified and collected data about women who gave birth in an AMU in the UK between 1 March 2017 and 28 February 2018, and whose baby was admitted to a neonatal unit, for at least 4 hours, within 48 hours of birth or before discharge home or who was stillborn or died within 48 hours of birth without admission to neonatal care (cases). We refer to this outcome as ‘neonatal admission or mortality’. Controls were the two women not meeting the case definition who gave birth in the same AMU immediately before each case.

### Data collection

We collected data using the UK Midwifery Study System (UKMidSS), a national research infrastructure covering all 123 AMUs in all four countries of the UK. UKMidSS midwife ‘reporters’ in each AMU reported cases, and the number of AMU births, in response to monthly emails, and entered anonymised data directly from medical records using study-specific forms in a secure web-based environment.^16^

We compared the number of deaths reported for this study with those reported to national perinatal surveillance (MBRRACE-UK)^17^ in AMU births over the same period. Where this identified potentially ‘missing’ deaths, not reported to UKMidSS, we contacted UKMidSS and MBRRACE-UK reporters in the relevant units to cross-check using MBRRACE-UK and UKMidSS ID numbers (no identifiers were disclosed). Where a previously unreported case was identified, data were collected as described above.

### Data

We considered maternal sociodemographic factors; pre-existing clinical characteristics and those arising during pregnancy and intrapartum and birth-related factors, as potential explanatory variables (online supplementary table S1) and collected the neonatal outcomes listed in online supplementary table S2.

10.1136/archdischild-2020-319099.supp1 Supplementary data

### Analysis

We estimated the incidence of neonatal admission or mortality (combined and separately) using the total reported births as the denominator, with 95% CIs.

We described characteristics of cases and controls, and neonatal outcomes for cases. We used unconditional logistic regression to investigate univariable associations between explanatory variables and the primary outcome, and built a multivariable model, calculating unadjusted OR and adjusted OR (aOR) with 95% CI. Conditional logistic regression was not used since cases and controls were ‘convenience matched’ on the basis of time of birth only.^18^ We used a prespecified conceptual framework approach to multivariable model building, adding variables to the model in stages from distal to proximal (maternal and pre-existing clinical characteristics first, followed by maternal clinical characteristics arising during pregnancy, maternal intrapartum factors and finally, birth-related factors). Variables were considered for inclusion in the model if p<0.05 in the univariable analysis, or if univariable analyses indicated that their association with the outcome was confounded by another variable. The contribution of each variable to the fit of the data to the model was tested for significance using the Wald test, and variables for which p<0.05 were retained in the model. We used robust variance estimation to allow for the clustering of women within units.

In a post hoc analysis, we compared the highest level of care received and reasons for admission in centres where the number of cases reported or the incidence of admission was at or above the 95th centile, with other centres, using the χ^2^ test.

Our approach to handling missing data, and sample size and power calculations are presented in online supplementary box S1.

We used Stata V.15SE for all analyses.^19^

## Results

### Response and incidence

All 123 AMUs in the UK participated (100% of eligible units), with a 99.8% response to monthly report requests.

**Figure 1:**
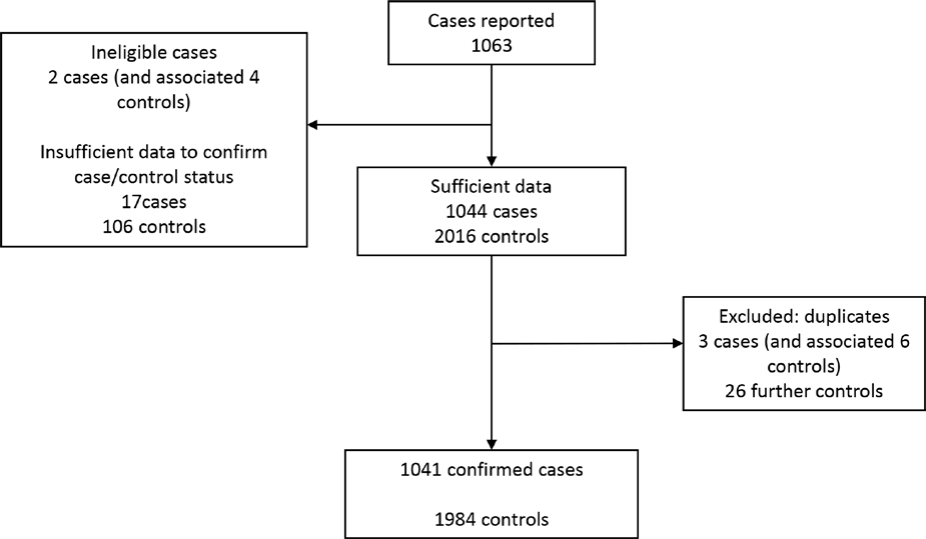
Reported and confirmed cases and controls with reasons for exclusion.

In total, 1063 cases were reported (figure 1). There were 1041 confirmed cases, and 1984 controls, from a total of 87 102 women giving birth in AMUs. Among the 1041 cases, two women had an intrapartum stillbirth and one woman’s baby died after birth without admission to neonatal care. Six of the 1038 surviving babies admitted to neonatal care subsequently died. The incidence of neonatal admission or mortality following birth in an AMU was 1.2% (95% CI 1.1 to 1.3), comprising neonatal admission (1.2%) and mortality (0.01%).

Overall, 110 out of 123 (85.4%) AMUs in the UK reported at least one confirmed case during the study period. The incidence of neonatal admission or mortality in each AMU ranged from 0% to 4.2% (median 1.0%; IQR 0.5%–1.6%).

### Univariable analysis

Among sociodemographic and pre-existing clinical characteristics of women, ethnicity, parity and previous pregnancy problems were statistically significantly associated with neonatal admission or mortality (table 1). Clinical characteristics arising during pregnancy that were statistically significantly associated with neonatal admission or mortality were: current pregnancy fetal problem and sex of the baby (table 1). Details of medical risk factors, and pregnancy problems are shown in online supplemental tables S3 and S4.

**Table 1:** Sociodemographic, pre-existing and pregnancy-related clinical characteristics of women

	Cases n=1041		Controls n=1984		Unadjusted analysis		
	n	%	n	%	OR	(95% CI)	P value
Maternal age (years)							0.30
Under 20	43	4.1	55	2.8	1.55	(0.98 to 2.46)	
20–24	177	17.0	325	16.4	1.08	(0.85 to 1.37)	
25–29	312	30.0	619	31.2	1		
30–34	342	32.9	628	31.7	1.08	(0.89 to 1.32)	
35–39	155	14.9	324	16.3	0.95	(0.74 to 1.22)	
≥40	12	1.2	33	1.7	0.72	(0.40 to 1.30)	
Missing	0		0				
Ethnic group							0.004
White (UK and Ireland)	641	61.6	1210	61.0	1		
White (other)	170	16.3	246	12.4	1.30	(1.05 to 1.62)	
Asian	140	13.5	289	14.6	0.91	(0.76 to 1.10)	
Black	51	4.9	134	6.8	0.72	(0.50 to 1.03)	
Other	39	3.8	105	5.3	0.70	(0.46 to 1.06)	
Missing	0		0				
Socioeconomic status							0.06
Higher managerial, admin, prof	301	28.9	540	27.2	1		
Intermediate	202	19.4	362	18.3	1.00	(0.81 to 1.24)	
Routine and manual	237	22.8	499	25.2	0.85	(0.69 to 1.06)	
Unemployed/Student	100	9.6	160	8.1	1.12	(0.88 to 1.43)	
Employed, job unrecorded or uncodable	66	6.3	123	6.2	0.96	(0.70 to 1.33)	
Employment status not recorded	135	13.0	300	15.1	0.81	(0.65 to 1.00)	
Area deprivation quintile*							0.29
First (least deprived)	175	17.0	374	19.0	1		
Second	192	18.6	390	19.9	1.05	(0.82 to 1.35)	
Third	211	20.5	409	20.8	1.10	(0.87 to 1.40)	
Fourth	242	23.5	408	20.8	1.27	(0.98 to 1.64)	
Fifth (most deprived)	210	20.4	383	19.5	1.17	(0.92 to 1.49)	
Missing	11		20				
Smoking status							0.71
Non-smoker during pregnancy	903	86.9	1720	86.7	1		
Smoker during pregnancy	125	12.0	235	11.8	1.01	(0.80 to 1.28)	
Not recorded	11	1.1	29	1.5	0.72	(0.32 to 1.61)	
Missing	2		0				
Previous pregnancies ≥24 weeks							<0.0001
0	523	50.2	704	35.5	1.98	(1.70 to 2.31)	
1	328	31.5	875	44.1	1		
2	137	13.2	280	14.1	1.31	(1.03 to 1.65)	
3 or more	53	5.1	125	6.3	1.13	(0.77 to 1.67)	
Missing	0		0				
BMI at booking (kg/m^2^)							0.44
<18.5	23	2.2	56	2.8	0.73	(0.42 to 1.26)	
18.5–24.9	529	50.8	1063	53.6	0.88	(0.75 to 1.05)	
25–29.9	296	28.4	526	26.5	1		
30–35.0	89	8.6	154	7.8	1.03	(0.76 to 1.38)	
>35.0	26	2.5	34	1.7	1.36	(0.78 to 2.37)	
Not recorded	78	7.5	151	7.6	0.92	(0.73 to 1.16)	
Missing	0		0				
Pre-existing medical risk factors†							0.69
None	1026	98.6	1956	98.7	1		
One or more	15	1.4	25	1.3	1.14	(0.59 to 2.21)	
Missing	0		3				
Problems in a previous pregnancy‡ (multiparous women only)							0.03
None	494	95.4	1247	97.4	1		
One or more	24	4.6	33	2.6	1.83	(1.05 to 3.20)	
Missing	0		0				
Current pregnancy maternal problem§							0.07
None	950	91.6	1847	93.4	1		
One or more	87	8.4	131	6.6	1.29	(0.98 to 1.71)	
Missing	4		6				
Current pregnancy fetal problem¶							0.03
None	1018	98.2	1960	99.1	1		
One or more	19	1.8	18	0.9	2.03	(1.06 to 3.88)	
Missing	4		6				
Sex of baby							<0.0001
Male	630	60.8	1009	50.9	1.50	(1.27 to 1.76)	
Female	406	39.2	973	49.1	1		
Missing	5		2				
Gestation at birth (weeks)							0.20
36–37	37	3.6	50	2.5	1.46	(0.91 to 2.35)	
38	127	12.3	213	10.8	1.18	(0.90 to 1.53)	
39	295	28.5	623	31.5	0.93	(0.78 to 1.12)	
40	362	34.9	714	36.1	1		
41–42	216	20.8	380	19.2	1.12	(0.94 to 1.34)	
Missing	4		4				

*Area deprivation quintiles created using children in low-income families measure.^37^

†Essential hypertension; confirmed cardiac disease; thromboembolic disorder; atypical antibodies; hyperthyroidism; diabetes; renal disease; epilepsy.

‡Unexplained stillbirth/neonatal death or previous death related to intrapartum difficulty; previous baby with neonatal encephalopathy; primary PPH requiring treatment/transfusion; shoulder dystocia; previous caesarean section; other.

§GBS; BMI >35 kg/m^2^; post-term pregnancy; pre-eclampsia/pregnancy-induced hypertension; preterm prelabour rupture of membranes; substance misuse/alcohol; gestational diabetes; other.

¶Malpresentation; small for gestational age; reduced fetal movements; fetal abnormality.

BMI, body mass index; GBS, group B streptococcus; PPH, postpartum haemorrhage.

Labour-related factors significantly associated with neonatal admission or mortality were: fetal complication at the start of labour care, stage of labour at admission, prolonged second stage of labour, immersion in water, opiates for pain relief, fetal heart rate concerns or other fetal complications identified during labour and consulting an obstetrician for fetal compromise (table 2). Details of complications are shown in online supplemental tables S5–S7. All four birth-related factors: shoulder dystocia, birth weight, birth in water and mode of birth were statistically significantly associated with neonatal admission or mortality (table 2).

**Table 2:** Labour and birth-related factors

	Cases n=1041		Controls n=1984		Unadjusted analysis		
	n	%	n	%	OR	(95% CI)	P value
Maternal complications identified at start of labour care*							0.30
None	1012	97.8	1943	98.3	1		
One or more	23	2.2	34	1.7	1.30	(0.79 to 2.13)	
Missing	6		7				
Fetal complications identified at start of labour care†							<0.0001
None	960	92.8	1908	96.5			
One or more	75	7.2	69	3.5	2.16	(1.52 to 3.06)	
Missing	6		7				
Stage of labour at admission							0.03
Latent	243	23.5	430	21.7	1.07	(0.90 to 1.26)	
Active first stage	709	68.5	1340	67.6	1		
Passive second stage	27	2.6	80	4.0	0.64	(0.43 to 0.94)	
Active second stage	56	5.4	131	6.6	0.81	(0.57 to 1.14)	
Missing	6		3				
Duration of first stage of labour‡							0.21
Within guidance	881	84.6	1706	86.0	1		
Possibly prolonged	20	1.9	21	1.1	1.84	(0.92 to 3.68)	
Not recorded	140	13.5	257	12.9	1.05	(0.85 to 1.31)	
Duration of second stage of labour§							0.02
Within guidance	882	84.7	1752	88.3	1		
Possibly prolonged	70	6.7	92	4.6	1.51	(1.06 to 2.15)	
Not recorded	89	8.6	140	7.1	1.26	(0.97 to 1.65)	
Immersion in water during labour							0.002
No	539	52.1	1152	58.2	1		
Yes	496	47.9	829	41.8	1.28	(1.10 to 1.49)	
Missing	3		3				
Pethidine/Diamorphine during labour							0.002
No	887	85.7	1779	89.8	1		
Yes	148	14.3	203	10.2	1.46	(1.16 to 1.85)	
Missing	6		2				
Duration between pethidine/diamorphine and birth (hours)¶							0.29
Mean (SD)	3.8	(2.4)	3.5	(2.6)	1.05	(0.96 to 1.15)	
Median (IQR)	3.3	(2.0 to 5.4)	2.9	(1.5 to 4.7)			
Missing (n)	22		39				
Fetal heart rate concerns identified							<0.0001
No	962	93.0	1942	98.0	1		
Yes	73	7.0	39	2.0	3.78	(2.62 to 5.45)	
Missing	6		3				
Maternal complications identified during labour (before birth)**							0.78
None	1019	98.5	1948	98.3	1		
One or more	16	1.6	33	1.7	0.93	(0.54 to 1.59)	
Missing	6		3				
Fetal complications identified during labour (before birth)††							<0.0001
None	798	77.1	1860	93.9	1		
One or more	237	22.9	121	6.1	4.57	(3.61 to 5.77)	
Missing	6		3				
Obstetrician consulted for maternal compromise during labour							0.58
No	1021	98.7	1959	98.8	1		
Yes	14	1.3	23	1.2	1.17	(0.67 to 2.03)	
Missing	6		2				
Obstetrician consulted for fetal compromise during labour							<0.0001
No	968	93.5	1925	97.1	1		
Yes	67	6.5	57	2.9	2.34	(1.63 to 3.35)	
Missing	6		2				
Shoulder dystocia							<0.0001
No	972	93.9	1958	98.8	1		
Yes	63	6.1	24	1.2	5.29	(3.27 to 8.55)	
Missing	6		2				
Birth weight (g)							<0.0001
<2500	25	2.4	12	0.6	4.65	(2.34 to 9.24)	
2500–2999	130	12.6	233	11.7	1.25	(0.95 to 1.63)	
3000–3499	383	37.0	855	43.2	1		
3500–3999	330	31.9	672	33.9	1.10	(0.90 to 1.33)	
4000–4499	137	13.2	183	9.2	1.67	(1.29 to 2.16)	
≥4500	31	3.0	26	1.3	2.66	(1.57 to 4.52)	
Missing	5		3				
Birth in water							0.01
No	789	76.2	1425	71.9	1		
Yes	246	23.8	557	28.1	0.80	(0.67 to 0.95)	
Missing	6		2				
Mode of birth							0.03
Spontaneous vertex	1014	98.0	1962	99.0	1		
Vaginal breech‡‡	8	0.8	1	0.1	15.48	(1.87 to 128.32)	
Instrumental	13	1.3	18	0.9	1.40	(0.62 to 3.13)	
Missing	6		3				

*Maternal tachycardia; hypertension; proteinuria; maternal fever; vaginal blood loss; prolonged membrane rupture; pain differing from contractions.

†Significant meconium; non-significant meconium; abnormal presentation; high/free floating head; suspected fetal growth restriction/macrosomia; suspected anhydramnios/polyhydramnios; fetal heart rate abnormality; fetal heart rate decelerations; reduced fetal movements in last 24 hours.

‡From start of active first stage to start of active second stage: within guidance ≤12 hours (nulliparous and multiparous); possibly prolonged >12 hours (nulliparous and multiparous).

§From start of active second stage to birth: within guidance ≤2 hours (nulliparous), ≤1 hour (multiparous); possibly prolonged >2 hours (nulliparous), >1 hour (multiparous).

¶In those who received pethidine/diamorphine.

**Maternal tachycardia; hypertension; maternal fever; vaginal blood loss; prolonged membrane rupture; pain differing from contractions.

††Significant meconium; confirmed/suspected first stage delay; confirmed/suspected second stage delay; obstetric emergency; abnormal presentation; transverse/oblique lie; high/free floating head; fetal heart rate abnormality; fetal heart rate decelerations.

‡‡All vaginal breech births were undiagnosed before admission. One woman had a precipitate birth, shortly after admission. In the remaining eight, abnormal presentation was noted on admission or during labour and five women were seen by an obstetrician in the AMU.

AMU, alongside midwifery unit.

### Multivariable analysis: factors associated with neonatal admission or mortality

Multivariable analysis identified white ‘other’ ethnicity; nulliparity; two or more previous pregnancies ≥24 weeks’ gestation; male sex; maternal current pregnancy problem (most common group B streptococcus (GBS) and body mass index (BMI)) >35 kg/m^2^, see online supplemental table S2); prolonged or unrecorded duration of second stage of labour; opiates during labour; shoulder dystocia and birth weight <2500 g, 4000–4999 g and ≥4500 g, as independently associated with neonatal admission or mortality (table 3).

**Table 3:** Factors associated with neonatal admission or mortality

	Cases n=1040		Controls n=1982		Unadjusted analysis		Adjusted analysis n=3002		
	n	%	n	%	OR	(95% CI)	aOR*	(95% CI)	P value
Ethnic group									0.01
White (UK and Ireland)	641	61.6	1210	61.0	1		1		
White (other)	170	16.3	246	12.4	1.30	(1.05 to 1.62)	1.28	(1.01 to 1.63)	
Asian	140	13.5	289	14.6	0.91	(0.76 to 1.10)	0.94	(0.76 to 1.16)	
Black	51	4.9	134	6.8	0.72	(0.50 to 1.03)	0.73	(0.51 to 1.04)	
Other	39	3.8	105	5.3	0.70	(0.46 to 1.06)	0.68	(0.44 to 1.05)	
Previous pregnancies ≥24 weeks									<0.0001
0	523	50.2	704	35.5	1.98	(1.70 to 2.31)	2.09	(1.78 to 2.45)	
1	328	31.5	875	44.1	1		1		
2 or more	190	18.3	405	20.4	1.25	(1.00 to 1.56)	1.38	(1.10 to 1.74)	
Sex of baby									<0.0001
Female	406	39.2	973	49.1	1		1		
Male	630	60.8	1009	50.9	1.50	(1.27 to 1.76)	1.46	(1.23 to 1.75)	
Current pregnancy maternal problem†									0.03
None	950	91.6	1847	93.4	1		1		
One or more	87	8.4	131	6.6	1.29	(0.98 to 1.71)	1.40	(1.03 to 1.90)	
Duration of second stage of labour‡									0.01
Within guidance	882	84.7	1752	88.3	1		1		
Possibly prolonged	70	6.7	92	4.6	1.51	(1.06 to 2.15)	1.42	(1.01 to 2.01)	
Not recorded	89	8.6	140	7.1	1.26	(0.97 to 1.65)	1.38	(1.05 to 1.81)	
Pethidine/Diamorphine during labour									0.04
No	887	85.7	1779	89.8	1		1		
Yes	148	14.3	203	10.2	1.46	(1.16 to 1.85)	1.31	(1.02 to 1.68)	
Shoulder dystocia									<0.0001
No	972	93.9	1958	98.8	1		1		
Yes	63	6.1	24	1.2	5.29	(3.27 to 8.55)	5.06	(3.00 to 8.52)	
Birth weight (g)									<0.0001
<2500	25	2.4	12	0.6	4.65	(2.34 to 9.24)	4.12	(1.97 to 8.60)	
2500–2999	130	12.6	233	11.7	1.25	(0.95 to 1.63)	1.20	(0.91 to 1.59)	
3000–3499	383	37.0	855	43.2	1		1		
3500–3999	330	31.9	672	33.9	1.10	(0.90 to 1.33)	1.10	(0.90 to 1.34)	
4000–4499	137	13.2	183	9.2	1.67	(1.29 to 2.16)	1.64	(1.25 to 2.14)	
≥4500	31	3.0	26	1.3	2.66	(1.57 to 4.52)	2.10	(1.17 to 3.76)	

*Adjusted for all other variables in the model.

†GBS; BMI >35 kg/m^2^; post-term; pre-eclampsia/pregnancy-induced hypertension; preterm prelabour rupture of membranes; substance misuse/alcohol; gestational diabetes.

‡From start of active second stage to birth: within guidance ≤2 hours (nulliparous), ≤1 hour (multiparous); possibly prolonged >2 hours (nulliparous), >1 hour (multiparous).

BMI, body mass index; GBS, group B streptococcus.

### Neonatal outcomes

Around three-quarters (78%) of the cases admitted to neonatal care were admitted from the birth room, rather than from the postnatal ward (table 4). Less than half (43%) received any neonatal resuscitation and in around 80% of those this comprised stimulation/positioning, inflation breaths and oxygen/ventilation breaths only. Among cases who were resuscitated, 17% were intubated and 2% received neonatal resuscitation drugs. Around one in five cases (18%) who were admitted to neonatal care received intensive care. The most common reasons for admission were respiratory problems and suspected infection, and this was reflected in diagnoses on discharge.

**Table 4:** Neonatal outcomes

	Cases n=1041		Controls n=1984	
	n	%	n	%
Apgar score <7 at 5 min				
Yes	222	21.5	10	0.5
Missing	7		2	
Neonatal resuscitation				
Yes	448	43.2	51	2.6
Missing	5		2	
Type of resuscitation (among those who received resuscitation)				
Stimulation	375	83.7	47	92.2
Positioning/Managing airway	362	80.8	27	52.9
Five inflation breaths	386	86.2	31	60.8
Oxygen	261	58.3	11	21.6
Ventilation breaths	277	61.8	10	19.6
Intubation	77	17.2	0	
Chest compression	32	7.1	1	2.0
Neonatal resuscitation drug	9	2.0	0	
Hierarchy of resuscitation (among those who received resuscitation)*				
Airway (A): stimulation/positioning only	15	3.4	17	33.3
Breathing 1 (B1): A or five inflation breaths	73	16.3	17	33.3
Breathing 2 (B2): A or B1 or oxygen/ventilation breaths	275	61.4	16	31.4
Breathing 3 (B3): A or B1 or B2 or intubation	51	11.4	0	
Chest compression (C): A or B or chest compression	25	5.6	1	2.0
Drugs: A or B or C or neonatal drugs	9	2.0	0	
Neonatal team consulted while baby in midwifery unit				
Yes	893	86.2	136	6.9
Missing	5		4	
Primary reason neonatal team consulted				
Respiratory problems	572	64.6	26	19.7
Suspected infection	31	3.5	16	12.1
Suspected perinatal asphyxia	89	10.1	14	10.6
Meconium aspiration	54	6.1	20	15.2
Congenital anomaly	18	2.0	12	9.1
Feeding problems	18	2.0	4	3.0
Physical trauma/birth injury	11	1.2	6	4.6
Other	93	10.5	34	25.8
Missing	7		4	
Age of baby when neonatal team first consulted (hours)				
Mean (SD)	4.5	(7.2)	6.9	(10.6)
Median (IQR)	0.6	(0.7 to 6.3)	2.2	(0.3 to 8.5)
Skin to skin				
Yes	885	86.1	1921	97.2
Missing	13		7	
Initiation of breast feeding				
Yes	794	77.0	1622	82.0
Missing	10		6	
Age of baby at neonatal admission (hours)	**n=1038**		**n=13†**	
Mean (SD)	6.4	(8.4)	10.1	(14.4)
Median (IQR)	2.3	(0.6 to 9.5)	2.1	(2.0 to 11.5)
Where was baby admitted from				
Birth room	802	77.7		
Postnatal ward	230	22.3		
Missing	6			
Highest level of care baby received				
Intensive care	182	17.7		
High dependency care	319	31.0		
Special care	527	51.3		
Missing	10			
Reasons for admission‡				
Respiratory problems	731	70.4		
Suspected infection	438	42.2		
Suspected perinatal asphyxia	102	9.8		
Meconium aspiration	86	8.3		
Hypoglycaemia	57	5.5		
Congenital anomaly	46	4.4		
Feeding problems	44	4.2		
Jaundice	32	3.1		
Cardiac problems	23	2.2		
Pulse oximetry	15	1.5		
Abnormal movements	14	1.4		
Hypothermia	13	1.3		
Physical trauma/birth injury	11	1.1		
Maternal substance abuse	6	0.6		
Other§	32	3.1		
Diagnoses on discharge‡				
Sepsis	536	51.9		
Respiratory distress syndrome	429	41.6		
Congenital pneumonia	41	4.0		
Transient tachypnoea of the newborn	64	6.2		
Pneumothorax	18	1.7		
Hypoxic ischaemic encephalopathy	74	7.2		
Meconium aspiration syndrome	62	6.0		
PPHN	13	1.3		
Hypoglycaemia	63	6.1		
Jaundice	68	6.6		
Feeding problems	30	2.9		
Birth injury	8	0.8		
Congenital anomaly	56	5.4		
Cardiac problems	20	1.9		
Neonatal abstinence syndrome/social	4	0.4		
Normal	16	1.6		
Insufficient information/not specified	78	7.6		
Other	17	1.6		

*Mutually exclusive hierarchy in which each category includes those babies who received that type of resuscitation, excluding those who also received any resuscitation type higher in the hierarchy.

†Thirteen babies in the control group were admitted to neonatal care for less than <4 hours. Other data for these 13 babies not shown because of small numbers.

‡More than one reason for admission/discharge diagnosis could be given.

§Other includes: shoulder dystocia, observation, skin rash, intrauterine growth restriction/low birth weight and reason not specified.

#### Centres with high numbers of cases

Compared with other centres, in centres with more cases or higher incidence, a higher proportion of babies received special care as the highest level of neonatal care (64% vs 48%, p<0.001), fewer babies were admitted because of respiratory problems (60% vs 73%, p<0.0001) and more babies were admitted with suspected infection (52% vs 40%, p=0.001) (online supplemental table S8).

## Discussion

Around 1% of babies born in UK AMUs during the study period were admitted to neonatal care, stillborn or died within 48 hours of birth without admission. Less than half of the babies admitted to neonatal care required resuscitation and in around 80% of those this comprised stimulation/positioning, inflation breaths and oxygen/ventilation breaths only. The most common reasons for admission were respiratory problems and suspected infection; this was reflected in discharge diagnoses. We found significant variation between units in rates and reasons for admission.

National statistics show that the overall incidence of neonatal admission in term babies is around 6%^1^ and the incidence of intrapartum-related stillbirth or neonatal death at term is 0.28 per 1000 total births.^20^ The lower incidence of these outcomes found in our study reflects the predominantly ‘low risk’ characteristics of the study population.

Babies of women with identified maternal pregnancy complications were 1.4 times more likely to be admitted to neonatal care. In line with other evidence,^7^ the most common of these complications were GBS colonisation and BMI >35 kg/m^2^. National guidance advises that women with a BMI >35 kg/m^2^, and those with GBS for whom antibiotics in labour would be recommended, should be advised to plan birth in an OU rather than a midwifery-led setting, the latter group so they can receive intrapartum antibiotic prophylaxis.^10 21^ Almost half of UK midwifery units now report admitting women who require antibiotics for GBS^9^ and admission of women with a BMI >35 kg/m^2^ is also widespread.^8^ ‘Higher risk’ women in AMUs are more likely to be transferred before birth than women at ‘low risk’ of complications, but around 70% of ‘higher risk’ women admitted to AMUs give birth there without transfer.^7^ Planned birth in an AMU is associated with a significant reduction in the likelihood of having a Caesarean section compared with planned OU birth,^6^ and there is evidence of good outcomes for women with higher BMI in AMUs.^8^ Individual care plans are recommended for women at ‘higher risk’ of complications who plan to give birth outside an OU.^10^

Even in term appropriately grown babies, boys are more likely than girls to have lower Apgar scores, need neonatal resuscitation, develop respiratory problems and be admitted for neonatal care.^22^ Our finding, that boys were almost 1.5 times more likely than girls to be admitted for neonatal care, in a population in which 99% of women had a spontaneous vaginal birth, suggest that these differences are not explained by an increased risk of instrumental or operative birth,^22^ and are independent of birth weight.

Evidence about the duration of labour and neonatal outcomes is mixed,^23^ but recent studies point to an increased risk of adverse neonatal outcomes with prolonged second stage of labour.^15 24 25^ We found that the likelihood of neonatal admission or mortality was 1.4 times higher when the second stage of labour was ‘possibly prolonged’.^10^ Given available data it was not possible to determine why these women were not transferred to an OU, as national guidance recommends, nor whether the outcome for the baby would have been different had transfer taken place. For some women, for example, free text comments indicated that when concerns were identified late in labour an obstetrician attended the AMU to expedite birth, in preference to transfer, but available data did not permit further investigation. This might also explain at least some of the 31 instrumental births in an AMU in our study. Free text comments also indicated that, for some women, fetal concerns such as significant meconium and fetal heart decelerations were only identified in advanced labour or close to the time of birth. This might explain why these woman were not transferred, as recommended by national guidance,^10^ however the extent to which this also explained the lack of consultation with an obstetrician, seen in several cases, was not clear.

Opioids are widely used for pain relief in labour,^26^ but are associated with neonatal respiratory depression.^27 28^ In line with other studies,^15 29^ we found that the likelihood of neonatal admission or mortality was 1.3 times higher in babies of women who used opioids for pain relief in labour.

Our study confirms low (<2500 g) and high (>4000 g) birth weight as independent risk factors for adverse neonatal outcome.^29–32^ Fetal macrosomia is a risk factor for shoulder dystocia.^33 34^ Almost half of the cases with shoulder dystocia in our study had a birth weight >4000 g, but shoulder dystocia was associated with a fivefold increase in the likelihood of neonatal admission after adjusting for birth weight. Shoulder dystocia is a risk factor for birth trauma injuries,^35^ but in our study only a small proportion of the babies with documented shoulder dystocia had a discharge diagnosis of birth injury.

Finally, there are national initiatives aimed at reducing unnecessary neonatal admissions, avoiding separation of mother and baby, and standardising admission criteria,^13 36^ and some evidence that the provision of transitional care is increasing.^4^ Our study provides further evidence of variation in local neonatal unit admission policies,^14^ with significant variation between units in admission rates and reasons for admission.

This was a national population-based study, which reduces the risk of bias associated with local, hospital-based studies. All eligible units participated, with over 99% response to monthly report requests and complete data returned for over 95% of reported cases, reducing the possibility of selection bias. There are nevertheless some potential limitations. We aimed to identify all women whose baby was admitted to neonatal care or who died following birth in an AMU. We checked against deaths reported to MBRRACE-UK, so are confident that we are unlikely to have missed deaths, but had no other sources of data against which to validate other reported cases, so it is possible that we may have missed some admissions. We were dependent on anonymised routine data from medical records so did not have data on a number of factors of interest, including, for example, staffing levels, time of day, day of the week or whether there was an agreed plan for care of the woman in an AMU in the presence of risk factors.

## Conclusions

The results of this study are broadly reassuring and in line with existing evidence about the quality and safety of care in AMUs. Relatively few babies had diagnoses of suspected asphyxia or meconium aspiration, which might be indicative of serious intrapartum-related adverse outcome. Many of the factors we identified as associated with neonatal admission or mortality are known risk factors for adverse neonatal outcome in term infants in other settings. Midwives should continue to practice in line with national guidance in relation to the management of risk factors and emerging complications in women labouring in AMUs.
