# Supplementary material for: Neonatal admission and mortality in babies born in UK alongside midwifery units: a national population-based case-control study using the UK Midwifery Study System (UKMidSS)
Source: Arch Dis Child Fetal Neonatal Ed. 2020 Oct 30;106(2):194–203. doi: 10.1136/archdischild-2020-319099 (PMC7907574; doi:10.1136/archdischild-2020-319099)
Supplement: Supplementary data [file archdischild-2020-319099supp001.pdf]

SUPPLEMENTARY INFORMATION

Table S1: Potential explanatory variables and categorisation

**Type of characteristic**<br>Variable (unit or how derived)	**Categorisation**
**Maternal social demographic**	
Maternal age (years)	Under 20; 20-24;25-29; 30-34; 35-39; ≥40
Ethnic group	White (UK & Ireland); White (other); Asian; Black; Other
Socioeconomic status (derived from woman's occupation, or partner's if woman not in work or with unrecorded/uncodable occupation, using three class NS-SEC)	Higher managerial, admin, prof; Intermediate; Routine and manual; Unemployed/student; Employed, job unrecorded or uncodable; Employment status not recorded
Area deprivation quintile (derived from women's postcode entered by midwives in to postcode look-up website which returned score from Children in Low-income Families Measure, converted into quintiles)	1st (least deprived); 2nd; 3rd; 4th; 5th (most deprived)
Smoking status	Non-smoker during pregnancy; Smoker during pregnancy; Not recorded
**Pre-existing clinical factors**	
Previous pregnancies ≥24 weeks' gestation	0; 1; 2; ≥3
Body mass index (BMI) at booking (kg/m^2)	<18.5; 18.5-24.9; 25-29.9; 30-35.0; >35.0; Not recorded
Pre-existing medical risk factors (see Table S3)	None; One or more
Problems in a previous pregnancy (see Table S3)	None; One or more
**Clinical factors arising during pregnancy**	
Maternal current pregnancy problem (see Table S4)	None; One or more
Fetal current pregnancy problem (see Table S4)	None; One or more
Sex of baby	Male; Female
Gestation at birth (weeks)	36-37; 38; 39; 40; 41-42
**Intrapartum-related**	
Maternal complications identified at start of labour care (see Table S5)	None; One or more
Fetal complications identified at start of labour care (see Table S5)	None; One or more
Stage of labour at admission	Latent; Active 1st stage; Passive 2nd stage; Active 2nd stage
Duration of 1st stage of labour[1]	Within guidance; Possibly prolonged; Not recorded[1]
Duration of 2nd stage of labour[2]	Within guidance; Possibly prolonged; Not recorded[2]
Immersion in water during labour	No; Yes
Pethidine/diamorphine during labour	No; Yes
Duration between pethidine/diamorphine and birth (hours)	Mean (SD); Median (IQR)
Fetal heart rate concerns identified	No; Yes

**Type of characteristic**<br>Variable (unit or how derived)	**Categorisation**
Maternal complications identified during labour, before birth (see Table S6)	None; One or more
Fetal complications identified during labour, before birth (seeTable S6)	None; One or more
Obstetrician consulted for compromise during labour (see Table S6)	No; Yes
**Birth-related**	
Shoulder dystocia	No; Yes
Birthweight (g)	<2500; 2500-2999; 3000-3499; 3500-3999; 4000-4499; ≥4500
Birth in water	No; Yes
Mode of birth	Spontaneous vertex; Vaginal breech; Instrumental

[1] From start of active 1st stage to start of active 2nd stage: Categorised using NICE CG190 as: Within guidance ≤12 hours (nulliparous and multiparous); Possibly prolonged >12 hours (nulliparous and multiparous)
[2] From start of active 2nd stage to birth: Categorised using NICE CG190 as: Within guidance ≤2 hours (nulliparous), ≤1 hour (multiparous); Possibly prolonged >2 hours (nulliparous), >1 hour (multiparous)

Table S2: Neonatal outcomes and categorisation

**Neonatal outcome (unit or how derived**	**Categorisation**
Apgar score at 5 minutes	≥7; <7
Neonatal resuscitation	No; Yes
Type of resuscitation	Stimulation; Positioning/managing airway; Five inflation breaths; Oxygen; Ventilation breaths; Intubation; Chest compression; Neonatal resuscitation drug
Neonatal team consulted while baby in midwifery unit	No; Yes
Primary reason for neonatal consultation	Respiratory problems; Suspected infection; Suspected perinatal asphyxia; Meconium aspiration; Congenital anomaly; Feeding problems; Physical trauma/birth injury; Other
Age of baby when neonatal team first consulted (hours)	Mean (SD); Median (IQR)
Skin to skin care	No; Yes
Initiation of breastfeeding	No; Yes
Age of baby at neonatal admission (hours)	Mean (SD); Median (IQR)
Where was baby admitted from	Birth room; Postnatal ward
Highest level of care baby received	Intensive care; High dependency care; Special care
Reasons for admission	Respiratory problems; Suspected infection; Suspected perinatal asphyxia; Meconium aspiration; Hypoglycaemia; Congenital anomaly; Feeding problems; Jaundice; Cardiac problems; Pulse oximetry; Abnormal movements; Hypothermia; Physical trauma/birth injury; Maternal substance abuse; Other
Diagnoses on discharge	Sepsis; Respiratory Distress Syndrome; Congenital Pneumonia; Transient Tachypnoea of the Newborn; Pneumothorax; Hypoxic Ischaemic Encephalopathy; Meconium Aspiration Syndrome; PPHN; Hypoglycaemia; Jaundice; Feeding problems; Birth injury; Congenital anomaly; Cardiac problems; Neonatal Abstinence Syndrome/Social; Normal; Insufficient information/not specified; Other

Approach for handling missing data

We anticipated that some of the data required to generate body mass index would be unrecorded in women's notes, and that this 'missing' data would not be randomly distributed, so we provided the option at data entry of indicating that height or weight were 'not recorded' and used this category in our analysis.

The only other variable with a substantial proportion of missing data was socioeconomic status. This occurred because women's or their partner's occupation is often not recorded in women's notes, and we know from previous work (e.g. Lindquist A, Knight M, Kurinczuk JJ. Variation in severe maternal morbidity according to socioeconomic position: a UK national case-control study. BMJ Open 2013;3:e002742 doi: :10.1136/bmjopen-2013-002742) that these data are not missing at random. Approaches such as multiple imputation would not therefore be appropriate. We therefore included a separate 'missing/not recorded' category in our analyses for these variables.

Sample size and power

We anticipated identifying approximately 900 cases over one year, based on an estimated incidence of 1.5% in a population of around 60,000 women (Hollowell J, Puddicombe D, Rowe R, et al. The Birthplace national prospective cohort study: perinatal and maternal outcomes by planned place of birth. Birthplace in England research programme. Final report part 4. London: NIHR Service Delivery and Organisation programme; 2011). The actual number of cases and controls generated 80% power at the 5% level to detect ORs of 1.6 or greater for an exposure variable with a frequency of 5%, and 1.3 or greater for an exposure variable with a frequency of 30%.

Box S1: Additional methodological detail

Table S3: Pre-existing medical and previous pregnancy risk factors

	Cases (n=1041)		Controls (n=1981)	
	n	%	n	%
**Medical risk factors**				
Essential hypertension	3	0.3	2	0.1
Confirmed cardiac disease	2	0.2	1	0.1
Thromboembolic disorder	0		5	0.3
Atypical antibodies	5	0.5	4	0.2
Hyperthyroidism	3	0.3	5	0.3
Diabetes	2	0.2	3	0.2
Renal disease	0		3	0.2
Epilepsy	0		2	0.1
**Problems in previous pregnancy**	**Multiparous women only** n=518		n=1280	
Previous unexplained/intrapartum-related stillbirth/death	0		3	0.2
Previous baby with Neonatal Encephalopathy	1	0.2	0	0.0
Previous primary postpartum haemorrhage requiring treatment	11	2.1	19	1.5
Previous shoulder dystocia	5	1.0	3	0.2
Previous Caesarean section	5	1.0	5	0.4
"Other" previous pregnancy problem	2	0.4	3	0.2

Table S4: Current pregnancy problems

	Cases (n=1037)		Controls (n=1978)	
	**n**	**%**	**n**	**%**
**Maternal**				
Group B Streptococcus	47	4.5	79	4.0
BMI>35kg/m^2	26	2.5	34	1.7
Post-term (>42 weeks)	4	0.4	6	0.3
Pre-eclampsia/pregnancy induced hypertension	3	0.3	1	0.1
Preterm prelabour membrane rupture	0		1	0.1
Substance misuse/alcohol dependency	4	0.4	7	0.4
Gestational diabetes	4	0.4	8	0.4
Other	1	0.1	1	0.1
**Fetal**				
Multiple birth	0		0	
Malpresentation	2	0.2	1	0.1
Small for gestational age	7	0.7	9	0.5
Reduced fetal movements	4	0.4	9	0.5
Fetal abnormality	6	0.6	0	

Table S5: Complications identified at start of labour care

Complications identified at start of labour care	Cases (n=1035[1])		Controls (n=1977[1])	
	**n**	**%**	**n**	**%**
**Maternal**				
Maternal tachycardia	9	0.9	7	0.4
Essential hypertension	2	0.2	4	0.2
Proteinuria	2	0.2	4	0.2
Maternal pyrexia	0		1	0.1
Vaginal blood loss	3	0.3	1	0.1
Prolonged membrane rupture	11	1.1	19	1.0
Pain differing from contractions	1	0.1	1	0.1
**Fetal**				
Significant meconium	14	1.4	10	0.5
Non-significant meconium	22	2.1	27	1.4
Abnormal presentation	3	0.3	0	
High /free floating head	1	0.1	0	
Suspected fetal growth restriction / macrosomia	5	0.5	4	0.2
Suspected anhydramnios / polyhydramnios	2	0.2	0	
Fetal heart rate abnormality	6	0.6	5	0.3
Fetal heart rate decelerations	9	0.9	6	0.3
Reduced fetal movements in last 24 hours	18	1.7	18	0.9

[1] Those with non-missing data for complications identified at start of labour care

Table S6: Complications identified during labour (before birth)

**Complications identified during labour (before birth)**	**Cases (n=1035)**		**Controls (n=1981)**	
	**n**	**%**	**n**	**%**
**Maternal**				
Maternal tachycardia	6	0.6	9	0.5
Hypertension	4	0.4	4	0.2
Maternal pyrexia	5	0.5	3	0.2
Vaginal blood loss	7	0.7	10	0.5
Prolonged membrane rupture	7	0.7	11	0.6
Pain differing from contractions	1	0.1	0	
**Fetal**				
Significant meconium	120	11.6	49	2.5
Confirmed/suspected 1st stage delay	14	1.4	11	0.6
Confirmed/suspected 2nd stage delay	12	1.2	10	0.5
Obstetric emergency	15	1.5	2	0.1
Abnormal presentation	7	0.7	2	0.1
High /free floating head	2	0.2	0	
Fetal heart rate abnormality	22	2.1	14	0.7
Fetal heart rate decelerations	74	7.2	48	2.4

Note: more than one complication could be identified in each woman

Table S7: Reasons for consultation with obstetrician during labour (before birth)

**Primary reason for consulting obstetrician during labour**	**Cases (n=82[1])**		**Controls (n=80[1])**	
	**n**	**%**	**n**	**%**
**Maternal**				
Maternal tachycardia	1	1.2	1	1.3
Hypertension	0		2	2.5
Maternal pyrexia	0		2	2.5
Vaginal blood loss	1	1.2	0	
Prolonged membrane rupture	1	1.2	4	5.0
Pain differing from contractions	0		1	1.3
Other maternal[2]	11	13.4	15	18.8
**Fetal**				
Significant meconium	5	6.1	5	6.3
Confirmed/suspected 1st stage delay	6	7.3	9	11.3
Confirmed/suspected 2nd stage delay	4	4.9	11	13.8
Obstetric emergency	14	17.1	1	1.3
Abnormal presentation	7	8.5	1	1.3
Fetal heart rate abnormality	13	15.9	8	10.0
Fetal heart rate decelerations	18	22.0	23	28.8
Reduced fetal movements	4	4.9	2	2.5
Other fetal[2]	4	4.9	3	3.8

[1] Those for whom an obstetrician was consulted during labour and a reason was given

[2] Other, including advising on suitability for AMU, categorised as maternal or fetal on individual basis

Note: More than one reason could be given for each woman

Table S8: Level of care and reasons for admission in centres with high and lower number of cases

	Centres with fewer cases (n=810)		Centres with more cases (n=228)		
	**n**	**%**	**n**	**%**	**p value**
**Highest level of neonatal care**					<0.0001
Intensive care	146	18.3	36	15.8	
High dependency care	272	34.0	47	20.6	
Special care	382	47.8	145	63.6	
Missing	10		0		
**Reasons for admission**					
Respiratory problems	594	73.3	137	60.1	<0.0001
Suspected perinatal asphyxia	81	10.0	21	9.2	0.72
Hypoglycaemia	40	4.9	17	7.5	0.14
Physical trauma/birth injury	9	1.1	2	0.9	0.76
Feeding problems	30	3.7	14	6.1	0.11
Suspected infection	320	39.5	118	51.8	0.001
Meconium aspiration	61	7.5	25	11.0	0.10
Jaundice	21	2.6	11	4.8	0.09
Congenital anomaly	34	4.2	12	5.3	0.49
Maternal substance abuse	6	0.7	0		0.19
Cardiac problems	18	2.2	5	2.2	1.0
Pulse oximetry	11	1.4	4	1.8	0.66
Abnormal movements	11	1.4	3	1.3	0.96
Hypothermia	11	1.4	2	0.9	0.56
Other	26	3.2	6	2.6	0.65
